# Evaluation of simplified wireless EEG recordings in the neurological emergency room

**DOI:** 10.1371/journal.pone.0310223

**Published:** 2024-10-31

**Authors:** Tamara M. Welte, Felix Janner, Sara Lindner, Stephanie Gollwitzer, Jenny Stritzelberger, Johannes D. Lang, Caroline Reindl, Maximilian I. Sprügel, David Olmes, Stefan Schwab, Christian Blinzler, Hajo M. Hamer

**Affiliations:** 1 Full member of ERN EpiCARE, Epilepsy Center, Department of Neurology, University Hospital Erlangen, Friedrich-Alexander University Erlangen-Nürnberg, Erlangen, Germany; 2 Department of Neurology, University Hospital Regensburg, Regensburg, Germany; Sorbonne Universite UFR de Biologie, FRANCE

## Abstract

**Objective:**

In the neurological emergency room (nER), timely electroencephalography (EEG) diagnostic is often crucial in patients with altered state of consciousness as well as in patients presenting with a first seizure. Yet, routine-EEG (rEEG) is often not available, especially during off-hours.

**Methods:**

We analyzed the value of a commercially available, simplified wireless eight-channel EEG recording (swEEG, CerebAir**®** EEG headset, Nihon Kohden), applied by non-EEG-specialized medical students, in patients presenting in our nER with (suspicion of) epileptic seizures and/or loss of or altered state of consciousness between 08/2019 and 08/2022. We evaluated the feasibility and validity compared to a standard rEEG (21 electrodes according to the international 10/20 system) and also included the clinical follow-up of the patients.

**Results:**

100 patients were included in our analysis (mean age 57.6 ± 20.4 years; 61 male). Median time of electrode application was 7 minutes (range 4–20 minutes), with significantly longer duration in patients with altered level of consciousness (median 8 minutes, p = 0.035). Electrode impedances also differed according to state of consciousness (p = 0.032), and were higher in females (p<0.001). 55 patients received additional rEEG, either during their acute nER stay (25) and/or during the next days (38). Considering normal EEG findings vs. pathological slowing vs. epileptiform activity, swEEG matched first rEEG results in 48/55 cases (87.3%). Overall, swEEG detected the same or additional pathological EEG patterns in 52/55 cases (94.5%). In 7/75 patients (9.3%) who did not receive rEEG, or had their rEEG scheduled to a later time point during their hospital stay, swEEG revealed important additional pathological findings (e.g. status epilepticus, interictal epileptiform discharges), which would have triggered acute therapeutic consequences or led to further diagnostics and investigations.

**Conclusion:**

The introduced swEEG represents a practicable, valuable technique to be quickly applied by non-EEG-specialized ER staff to initiate timely diagnostic and guide further investigations and treatment in the nER. Moreover, it may help to avoid under-diagnostic with potentially harmful consequences caused by skipped or postponed regular 10/20 EEG examinations, and ultimately improve the outcome of patients.

## Introduction

In the neurological emergency room (nER), timely electroencephalography (EEG) recordings are often recommended, e.g. in patients with altered state of consciousness to exclude status epilepticus and initiate treatment as soon as possible. In addition, it is a well-known fact that seizures are among the most frequent diagnoses in (n)ERs [1–6] with EEG recordings being a necessary part of the routine diagnostic in patients presenting with a first seizure [7]. Ideally this EEG should be obtained within 24 hours after the seizure to increase its sensitivity for interictal epileptiform activity (IED) [8, 9]. In patients with loss of consciousness due to unknown cause, EEG may also be applied as part of the evaluation to differential diagnosis of epileptic vs. non-epileptic events, e.g. syncope. Therefore, a 24/7 availability of EEG recordings should be aimed for at least in specialized neurological ERs (nERs).

Yet, EEG appears to be often underused in the emergency setting. In a recent nER analysis of patients presenting with seizures, EEG was only applied in less than one fifth of patients without known epilepsy, although it revealed meaningful results in a considerable subset of these patients [2]. This may be due to a lack of time, capacity, and specialized EEG technicians, especially during off-hours.

Therefore, there have been efforts in recent years to lower the hurdles for EEG recordings in the ER settings. One possible way to overcome the obstacles could be the application of a simplified EEG recording with a reduced number of electrodes which can be applied by ER staff not specialized in routine EEG recordings. The CerebAir® EEG headset by Nihon Kohden is one of these applications. It was initially developed for simplified continuous EEG monitoring in intensive care unit (ICU) settings, where its feasibility and validity has been evaluated already [10–12]. However, the feasibility and value of such EEG recordings have not yet been studied in an ER setting so far, nor in larger patients cohorts.

In this study, we aimed to evaluate the use of a simplified wireless EEG recording technique (swEEG) applied by non-EEG-specialized staff in patients presenting in the neurological emergency room. In addition, swEEG was compared to the standard procedure of routine-EEG (rEEG) recordings in the clinical setting.

## Methods

### Study site and patients

The study was conducted in the neurologist-led, specialized nER at the University Hospital Erlangen, with an approximate catchment area of one million inhabitants and >7000 nER visits per year. The nER provides a 24/7 neurological service with full access to main diagnostics (e.g. cerebral imaging, laboratory, etc.), and all specialized therapeutic procedures, including medical and endovascular interventions. Yet, availability of regular rEEG performed by specialized EEG technicians is limited to regular working hours (8am-4pm) without coverage of weekends and public holidays. For patients requiring hospitalization, the neurology department offers 80 standard care ward beds, a stroke/intermediate care unit (STU/IMC) and a neurologist-led specialized neurological intensive care unit (NICU). In general, rEEG examinations are performed in the EEG lab for both nER and hospitalized patients, according to the current guidelines of the German Society of Clinical Neurophysiology (21 electrodes according to the international 10/20 system, impedances <5kOhm, recording duration 20 minutes, www.dgkn.de).

For the current study, we included patients >18 years presenting in our nER with (suspicion of) epileptic seizures and/or loss of consciousness due to unknown cause, as well as patients with persistent altered state of consciousness at the time of admission to the nER. Study period was 1^st^ Aug 2019 – 31^st^ Aug 2022, with interruption for several months in 2020 and 2021 due to the COVID-19 pandemic. Data were analyzed retrospectively between 1^st^ Dec 2022 – 31^st^ May 2023 by TMW, HMH, FJ, and SL, who had access to individual and personalized data of study participants during and after data collection. The study was approved by the local ethics committee of the Friedrich-Alexander-University Erlangen-Nürnberg. All patients (when awake) or their relatives/legal representatives (when altered state of consciousness was still present) provided written informed consent for swEEG recording and for potential follow-up by telephone interviews before entering the study.

### EEG recording with CerebAir® EEG headset

After meeting the inclusion criteria and after receiving informed consent by the patients themselves or their relatives/legal representatives, the patients underwent swEEG. For the recording we used a commercial available CerebAir® EEG headset from Nihon Kohden which has already been described in previous studies [10, 11]. CerebAir® EEG headset, bedside EEG laptop with EEG review software ‘polaris.one’ and consumables (incl. EEG electrodes) were provided free of charge by Nihon Kohden Europe, Rosbach, Germany. There was no further funding involved. Nihon Kohden did not influence the idea and conduct of the study nor the results, their interpretation and publication.

For the swEEG recording, 7 pre-coated gel electrodes had to be attached to the headset, which then was placed on the patients head and fixed with soft bands. This resulted in electrode positions roughly covering Cz, F3, F4, T3, T4, C3, and C4 according to the International 10/20 System. Additionally, the headset offers the possibility to connect two additional cup electrodes to be fixed on the patient’s head with specific electrode paste. These were used to cover the parietooccipital regions aiming for positioning at O1 and O2, according to the international 10/20 system. Data were then transferred via bluetooth to the bedside EEG laptop where the data could be reviewed and were stored.

The swEEG recordings were performed autonomously by two non-EEG-trained medical students (FJ/SL) after an initial briefing by a technician of Nihon Kohden and an introduction to the principles of the International 10/20 system of EEG electrode placement by one of the authors (TMW). Availability of swEEG was not limited to regular working hours but was also possible during off-hours, including after hours and weekends. Concerning recording modalities, we aimed for a minimum of 10 minutes of EEG recording and applied stimulation maneuvers (eyes open/eyes closed and/or acoustic stimuli). The two medical students performing the swEEG recordings kept notes on problems occurring during application of the EEG device and recording of the EEG. swEEG was conducted only in agreement with nER staffing not to postpone other acute diagnostics and interventions. The rEEG review was not impacted by the study and was done as part of the routine assessment in the department of neurology by specialized neurology residents supervised by senior board certified EEG experts. The swEEG review was not performed on bedside but later by a resident of the epilepsy center (TMW), supervised by an EEG expert (HMH). At the time of review, both routine and swEEG reviewers were blinded for the EEG results of one another. For the review, swEEG was analyzed in a reduced bipolar montage (F3/C3, C3/O1, F3/T3, T3/O1 and F4/C4, C4/O1, F4/T4, T4/O2 respectively), or a reference montage using Cz as reference electrode.

To evaluate potential impact of the timely applied simplified EEG diagnostic, we followed-up patients’ medical records of the acute hospital stay as well as re-admissions. This was complemented by short telephone interviews 6 months after the initial nER stay in a subset of patients where a definite clinical diagnosis could not be established but swEEG had revealed pathological findings.

### Analysis and statistics

In the current study, the aim was to evaluate the feasibility and value of swEEG in a nER setting. Therefore, examined parameters included the duration for placement of the headset and electrodes, as well as electrode impedances as a marker of the quality of EEG recording. To evaluate the validity of swEEG, we compared the results to patients’ regular 10/20 EEG recordings, either obtained during nER stay, or at a later time point during their hospital stay. Results were classified as “normal”, and “pathological”. The latter category comprised “pathological slowing” (also distinguishing “focal” vs. “generalized slowing”), and “epileptiform activity” (comprising the variables “interictal epileptiform discharges”, “seizure patterns”, “status patterns”). In patients who had several rEEG recordings, comparison was always done with the first rEEG obtained.

As this was an explorative study, we mainly used descriptive statistics, and applied a non-parametric test (Mann-Whitney U test) for comparing swEEG and rEEG by the non-categorical variables of duration of electrode application (minutes), and electrode impedances (kOhm). The significance level was set to p<0.05.

## Results

### Patients and diagnoses

Among 101 swEEG recordings performed, 100 patients could be included in our analysis (mean age 57.6±20.4 years; 61 male, Table 1). One EEG recording was lost due to a failure of data storage.

**Table 1:** Patients’ characteristics.

n = 100	rEEG (n = 55)	No rEEG (n = 45)
Female (%)	18 (32.7)	21 (46.7)
Age (mean±SD)	61±19	53±21
Known diagnosis of epilepsy (%)	12 (21.8)	12 (26.7)
Prior history of seizures (%)	9 (16.3)	2 (4.4)
Awake at time of EEG (%)	33 (60.0)	34 (75.6)
Hospitalized in department of neurology (%)	46 (83.6)	10 (22.2)

rEEG = standard routine electroencephalography, SD = standard deviation

Of the 100 analyzed nER patients, 24 had a known diagnosis of epilepsy, and 11 patients had already one prior seizure documented, whereas 65 patients had no documented history of seizures nor epilepsy. 37 patients were discharged home after nER work-up, 56 were hospitalized in our department of neurology, and 7 were transferred to other departments of the hospital. The most common diagnosis at discharge was “epileptic seizure” (72 patients), including 5 (6.9%) patients with status epilepticus. Other diagnoses at discharge comprised syncope (10 patients), non-epileptic/dissociative seizures (2 patients), intoxication (2 patients), delirium associated to dementia/psychiatric disorder (2 patients), stroke (2 patients), post-commotio syndrome (2 patients), and 1 patient each with migraine with aura, infection/sepsis, pulmonary embolism, metabolic disorder, dysaesthesia/hypaesthesia of unknown cause. 3 patients were documented with loss of or altered consciousness due to unknown cause.

Overall, 67 patients were awake during swEEG recording, 12 patients were documented with confusion, 20 patients with somnolence and in 1 patient the EEG was recorded during coma and endotracheal intubation.

### Feasibility of swEEG

Median duration of electrode and headset application was 7 minutes (range 4–20). The time needed for electrode and headset application was longer in patients with altered level of consciousness (awake: median 7 min (range 4–12) vs. somnolent/comatose or agitated patients: median 8min (range 4–20). The mean impedance of electrodes was 36.3±13.3kOhm (n = 78 patients, as in n = 22 patients impedances were lost due to failure of data storage). Electrode impedances were significantly lower excluding the additional occipital electrodes (31kOhm vs. 37kOhm, p = 0.009), in male vs female patients (32kOhm vs. 41kOhm, p<0.001), and awake vs non awake patients (median 29kOhm vs. 40kOhm, p = 0.032).

Problems most frequently reported during application of swEEG comprised long/thick hair, lack of cooperation of patients with disturbed consciousness or agitation and high impedances especially of the additional parieto-occipital electrodes. This resulted in the need for rearrangement of the headset and/or application of additional EEG electrode conductive gel in 74 cases.

### Comparison of swEEG and routine EEG

55 patients received additional rEEG, either during their acute nER stay (n = 25) and/or during the following 1–3 days when hospitalized (n = 38, Table 1).

Comparing the categories of normal EEG findings vs. pathological slowing vs. epileptiform activity, swEEG matched rEEG results in 48/55 cases (87.3%, Table 2).

**Table 2:** Overview of rEEG and corresponding swEEG results.

		rEEG		
		normal	pathological slowing	epileptiform activity
swEEG	normal	22	0	0
	pathological slowing	4	15	1
	epileptiform activity	0	2	11

Matching results are displayed in grey. rEEG = standard routine electroencephalography, swEEG = simplified wireless electroencephalography (CerebAir®)

22/26 rEEG recordings classified as “normal”, also showed normal swEEG results (84.6%). In the remaining 4 patients with normal rEEG recordings (2 recorded during nER visit, 2 during further hospital stay), swEEG showed intermittent generalized slowing. Detailed swEEG results matched specific rEEG results in 45/55 cases (81.2%). Overall, swEEG detected the same or additional pathological EEG patterns in 52/55 cases (94.5%). The remaining 3 cases included one patient with focal and generalized slowing in rEEG, but only generalized slowing in swEEG. In another two patients, rEEG detected epileptiform activity, including one patient with a seizure and one patient with a status pattern, which in swEEG revealed only a continuous generalized slowing and interictal epileptiform discharges respectively (Table 3). In both cases, swEEG was performed after rEEG (>5 hours and >1hour later).

**Table 3:** Epileptiform activity in rEEG and corresponding swEEG results.

		rEEG		
		IED	Seizure	SE
swEEG	pathological slowing	0	1	0
	IED	4	0	1
	Seizure	1	1	0
	SE	0	0	4

Matching results are displayed in grey. IED = interictal epileptiform discharges, rEEG = standard routine electroencephalography; SE = status epilepticus, swEEG = simplified wireless electroencephalography (CerebAir®)

swEEG detected additional epileptiform EEG patterns in 3/55 cases. These included 2 patients with a seizure pattern in swEEG, which in their nER rEEG only showed IEDs (lateralized periodic discharges, LPD, Table 3), and a focal slowing (Table 2) respectively. The latter nER rEEG was the only one performed after swEEG (2,5 hours). Because of recurrent clinically evident focal seizure activity in nER, both patients were treated with intensified regimen of antiseizure medication (ASM). In a third patient with known epilepsy due to a prior intracerebral hemorrhage, swEEG detected IED, whereas rEEG the next day only revealed a focal slowing (Table 2). Although the presented 3 cases with additional pathological swEEG results could have had clinical implications in different clinical constellations, they did not result in an acute change of nER management in the individual clinical cases mentioned above.

### swEEG findings in patients without rEEG and potential benefits of the timely applied simplified EEG diagnostic in patients without or postponed rEEG diagnostic

Overall, 75 patients did not receive rEEG (n = 45), or had their rEEG scheduled to a later time point during their hospital stay (n = 30). Of those who did not receive rEEG 17/45 (37.8%) had normal swEEG findings, 19/45 (42.2%) had pathological slowing (10 generalized, 9 focal), and 9/45 (20.0%) showed epileptiform activity (8 IEDs, 1 status epilepticus).

Overall, within the group without or postponed rEEG examinations, there were 7/75 patients (9.3%, marked # throughout the text), where swEEG results would have triggered acute therapeutic consequences and/or led to further diagnostics and investigations.

**Fig 1:**
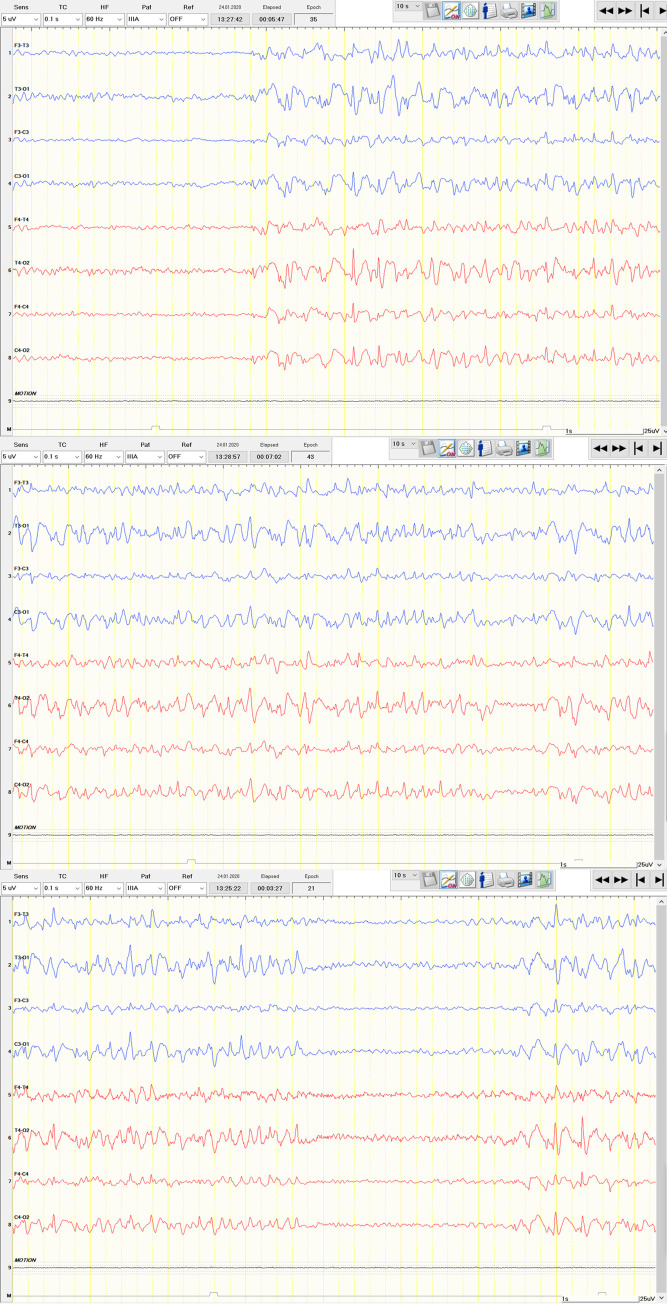
Absence status epilepticus. 42-year-old male with a known diagnosis of generalized epilepsy and intermediate disturbed consciousness. Simplified wireless EEG recording revealed absence status epilepticus.

Of the 9 patients who did not receive rEEG but had epileptiform activity in swEEG, 5 had a known diagnosis of epilepsy. One of them (42-year-old male #) with a known diagnosis of generalized epilepsy presented with intermediate disturbed consciousness. Under suspicion of intermittent absences he was sent home with slight increase of ASM (increase of levetiracetam from 1500mg/d to 1750mg/d with keeping dosage of lamotrigine at 400mg/d). swEEG during that nER visit already revealed evident absence status epilepticus (Fig 1). The patient then presented 3.5 weeks later in clinically worsened condition with disturbed consciousness, when rEEG ultimately confirmed absence status epilepticus, resulting in hospitalization and status treatment.

In the 4 cases showing IEDs in swEEG but without known diagnosis of epilepsy, first diagnosis of epilepsy was established in 2 patients with a first seizure because of cerebral imaging (prior intracerebral hemorrhage/first diagnosis of glioblastoma multiforme). In the remaining 2 patients, however, swEEG results would have resulted in further investigations.

**Fig 2:**
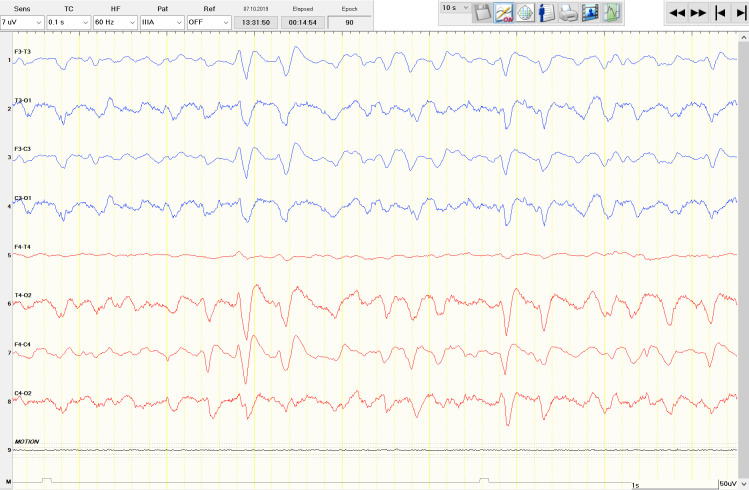
Generalized periodic discharges (GPDs) with triphasic morphology. 69-year-old female with a known history of hepatic failure and intermittent altered state of consciousness. Simplified wireless EEG recording showed GPDs with triphasic morphology consistent with a severe metabolic encephalopathy.

One of them was a 69-year-old female (#), who presented in the nER with a known history of hepatic failure and intermittent altered state of consciousness. As there was no obvious focal neurological sign at the time point of examination, and liver values were comparable to previous measurements (ammoniac was not tested), she was discharged home. swEEG at that time point already revealed continuous generalized slowing with generalized periodic discharges (GPDs) with triphasic morphology (Fig 2) [13], consistent with a severe metabolic encephalopathy. Four days later, the patient was again admitted to the nER in clinically worsened condition of sopor/coma. Ammoniac was then measured and was elevated to 417 μg/dl (ref: <82), she was then transferred to the medical intensive care unit.

The other patient, a 51-year-old male (#) presented with intermittent dysarthria and disturbed consciousness and was sent home after normal neurological examination and CT scan. swEEG at that time point revealed focal slowing right temporal with suspicion of IEDs. Six months later, he presented with the same complaints again and was hospitalized. Routine EEG only showed generalized slowing, but clinical suspicion of epileptic seizures was raised and ASM with levetiracetam was initiated.

In another two patients (29-year-old #, and 86-year-old males #) who did not receive rEEG and had suspected nER diagnosis of syncope after unobserved loss of consciousness, swEEG also revealed pathological results with continuous generalized slowing in both cases, as well as intermittent left focal slowing in the younger patient. In both patients outcome remains unclear, as they did not present again in our nER, and were not contactable via phone.

**Fig 3:**
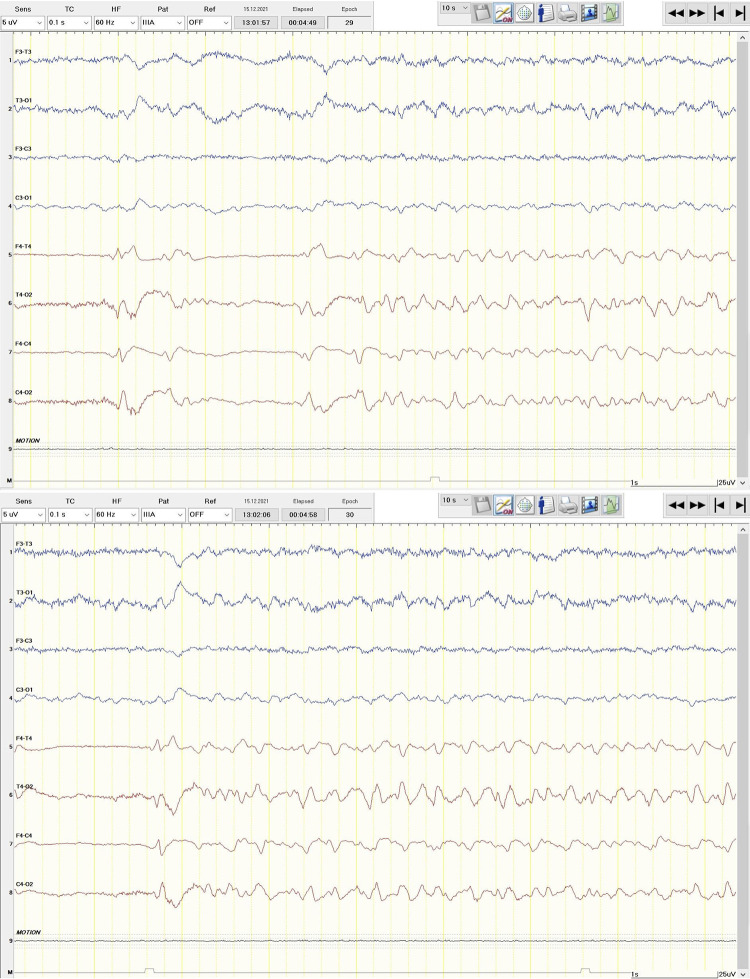
Focal status epilepticus. 70-year-old male with altered state of consciousness after (focal to) bilateral tonic-clonic seizure. Simplified wireless EEG recording revealed right focal status epilepticus.

In addition, 2 patients (45-year-old female #, and 70-year-old male #) received a rEEG later during their hospital stay but presented at the nER with altered state of consciousness after (focal to) bilateral tonic-clonic seizures and showed evidence of status epilepticus in the swEEG in the nER (Fig 3). However, it was not before the confirmation of status epilepticus in the rEEG that status treatment was initiated.

## Discussion

In this study, we evaluated the usefulness of a simplified wireless eight-channel EEG recording in a neurological emergency room and applied this EEG technique in a larger group of patients than in previous studies [10–12]. Our results suggest that such a device can be quickly applied by non-EEG-specialized ER staff, when rEEG is not readily available, and can generate meaningful information guiding further investigations and treatment in the nER. However, swEEG lacks the quality and accurateness of a standard rEEG which, therefore, should not be substituted by swEEG in the majority of the cases.

Overall, in our study the swEEG device was fast to apply with a median duration of electrode application of 7 minutes, which is comparable to previous investigations, and even shorter than rEEG application by expert staff reported in a previous study (swEEG 6 minutes vs. rEEG 10 minutes) [10]. However, one has to keep in mind the reduced subset of electrodes compared to rEEG. Similar to regular EEG recordings, swEEG struggles with certain problems affecting EEG quality, such as long/thick hair (especially in females) and lack of cooperation in patients with disturbed consciousness. Additionally, more difficult application of the additional occipital electrodes compared to the pre-coated gel electrodes was observed. These challenges could be overcome by e.g. re-application of electrode gel to finally gain analyzable EEG files. Yet, application of the device by non-EEG-specialized staff may have negatively affected recording quality, e.g. as mean electrode impedances were >10kOhm.

Nevertheless, considering the faster positioning without need for an EEG technician, together with the additional time saved normally needed for transportation to the EEG lab, makes the headset an ideal application for rapid nER assessment. Despite concerns with regard to validity of reduced EEG montages with limited sensitivity to detect epileptiform activity compared to standard EEG recordings in previous studies [14], simplified EEG recordings are more and more in use in (N)ICU settings [10–12, 15–18] as well as in (prehospital) emergency care [19–21]. Especially in the latter one, there is a need for rapid point-of-care testing to identify and guide further management of critically-ill patients. So far, however, most studies have been conducted in (N)ICU settings [10–12, 17, 18]. Therefore, reduced montage approaches are now increasingly investigated in tailored ER studies, using different EEG devices, ranging from 2-channel EEG to 10-electrode EEG headbands [22–24]. To our knowledge, the presented CerebAir® headset has not been investigated in an ER setting so far. Although many point-of-care EEG studies, similar to our study, report the difficulty of electrode placement and obtaining good EEG quality in patients with long/thick hair [19, 22], none of the present ER studies analyzed EEG quality systematically with e.g. impedances, nor reported detailed information on artefacts or EEG quality in general [22–24]. One study applying the CerebAir® device in an ICU setting reported a high number of interventions necessary for obtaining good impedances of EEG recordings assembled by a trained ICU physician [10], which is comparable to our observations. Some studies tried to solve the issue of reduced EEG quality in hairy regions by e.g. only choosing sub-hairline electrode locations, at the expense of reduced coverage and potentially reduced sensitivity to detect epileptiform activity [19]. Coming investigations will hopefully help to solve the questions around number and positioning of electrodes, as well as subtypes of electrodes (e.g. wet-gel vs. dry electrodes) for finding the optimal compromise of easy-to-use applications and diagnostic accuracy.

The swEEG recording as applied in the current study showed a good detection rate of pathological EEG findings also compared to rEEG, which is in line with a previous investigation [11]. In addition, in almost 10% of patients who did not receive rEEG or had their rEEG much later scheduled during their hospital stay, swEEG revealed results which would have triggered acute therapeutic consequences or led to further diagnostics and investigations, including status epilepticus, as well as pathological EEG findings in patients with unclear loss of or altered state of consciousness. This is in line with previous point-of-care EEG studies in ERs [22–24]. Consequently, our findings support the presence of a 24/7 availability in the nER to clarify diagnosis and initiate further diagnostic or treatment as soon as possible. Yet, the reality in (n)ERs can be quite different, with EEG diagnostic limited only to a small number of patients, and postponed or skipped rEEG examinations in many cases [2–4, 6].

However, our study was not intended and did not show that swEEG can substitute routine EEG recordings. The findings rather support the view that swEEG is a feasible and quickly to apply method to receive meaningful results when rEEG is not readily available. Therefore, swEEG may be seen as additional, quick ‘bedside’ test in the nER when sensitive time windows have to be considered, such as detection of IED after seizures for early specialized management and establishment of epilepsy diagnosis, which is crucial for patients’ outcomes [8, 9, 25], or treatment decisions regarding status epilepticus. Usually, a later routine EEG will have to follow with its better quality, longer recording time and higher spatial resolution. A swEEG should obviously not be used to safely localize IED or status epilepticus. Moreover, normal findings of swEEG should not be used to rule out epilepsy similarly to rEEG. The swEEG diagnostic will mostly focus on detection of pathological epileptiform activity such as IED or status patterns. rEEG will nevertheless remain the gold-standard.

In general, our findings also support the view, that repeated and/or longer EEG recordings are beneficial in catching pathological EEG activity [11, 26, 27]. This is underpinned by two cases where additional swEEG after rEEG ultimately revealed seizure activity, whereas rEEG in the acute setting only showed lateralized periodic discharges and a focal slowing respectively. This could be an advantage for applying the swEEG headset, which is also designed for long-term monitoring, and could be kept on the patient’s head during his/her entire stay at the nER in contrast to scheduled rEEG examinations, which are usually limited to 20 minutes and are usually performed in an EEG lab outside the nER.

Overall, simplified EEG applications such as used in this study can be applied by non-EEG-specialized staff and may contribute to overcome some of the EEG challenges such as time-constraints and limitations in capacity, especially during off-hours. In addition, such recordings enable timely rudimentary EEG diagnostic and consecutive work-up in the nER. However, interpretation of EEG data is at least equally important. It was not subject of this study to evaluate the ability of neurological ER staff to handle EEG recordings in a reduced montage. This warrants further studies likewise the question whether or not automated IED or seizure detection tools could be of special use in the emergency setting [28, 29]. Additionally, in the coming years, machine learning approaches will be of growing importance [21, 30].

The study also suffers from several limitations. First of all, the study population consisted in an unselected ER patient group with varying indication for an EEG recording, and because of the non-interventional and uncontrolled design, we cannot really measure the impact of swEEG on the course of the disease and the outcome of the patients. This should be addressed by future studies. Moreover, the comparability of swEEG to rEEG was hampered by the time delays of several hours or even a few days and also by the fact that only a subset of patients received both, swEEG and rEEG.

## Conclusion

In conclusion, timely EEG diagnostic is often crucial in the nER, especially in patients with altered state of consciousness as well as in persons presenting with a first seizure. The findings showed that swEEG is a feasible and quickly to apply method to receive meaningful results when rEEG is not readily available. Therefore, swEEG could serve as an additional, quick ‘bedside’ test in the nER when sensitive time windows have to be considered. However, it should not be used to substitute rEEG.
